# Source gene composition and gene conversion of the AluYh and AluYi lineages of retrotransposons

**DOI:** 10.1186/1471-2148-9-102

**Published:** 2009-05-14

**Authors:** Pamela Styles, John FY Brookfield

**Affiliations:** 1 Institute of Genetics, School of Biology, University of Nottingham, Nottingham, UK

## Abstract

**Background:**

Alu elements are a family of SINE retrotransposons in primates. They are classified into subfamilies according to specific diagnostic mutations from the general Alu consensus. It is now believed that there may be several retrotranspositionally-competent source genes within an Alu subfamily. In this study, subfamilies falling on the AluYi and AluYh lineages, and the AluYg6 subfamily, are assessed for the presence of secondary source genes, and the influence of gene conversion on the AluYh and AluYi lineages is also described.

**Results:**

The AluYh7 and AluYi6 subfamilies appear to contain multiple source genes. The novel subfamilies AluYh3a1 and AluYh3a3 are described, for which there is no convincing evidence to suggest the presence of secondary sources. The mutational substructure of AluYh3a3 can be explained completely by inference of single master gene. A complete backwards gene conversion event appears to have inactivated the AluYh3a3 master gene in humans. Polymorphism data suggest a larger number of secondary source elements may be active in the AluYg6 family than previously thought.

**Conclusion:**

It is clear that there is considerable variation in the number of source genes present in each of the young Alu subfamilies. This can range from a single master source gene, as for AluYh3a3, to as many as 14 source elements in AluYi6.

## Background

Alu elements, a family of non-autonomous SINE retrotransposons in primates, are approximately 300 bp in length, and have proliferated to over one million copies [1] in the human genome, comprising approximately 11% of the genome by mass [2]. Alu elements comprise two similar monomers derived from 7SL RNA in the common ancestor of primates and rodents [3].

The majority of these elements were generated 35–60 million years ago (mya) during the peak of Alu retrotranspositional activity [2], which has subsequently reduced to the current, relatively low level. Despite their high copy number, only a relatively small number of Alu elements are capable of generating new copies [4]. This has led to the generation of a collection of Alu subfamilies of differing ages, characterised by diagnostic mutations [5]. These correspond to mutations present within the source genes that gave rise to each subfamily.

The term "source gene" is used to describe an Alu element which is both transcriptionally and retrotranspositionally active, and therefore capable of producing daughter elements. For a long time, Alu elements were believed to follow a master gene pattern of expansion [2], whereby only one, or very few, elements are retrotranspositionally competent. Consequently, elements were grouped into subfamilies, elements within which share diagnostic mutations inferred to have occurred within the master gene. Currently, Alu elements are grouped into three groups of differing ages, AluJ, AluS and AluY. The elements derived from AluY, as discussed here, are still actively retrotransposing, and are therefore less divergent than the other groups. Subfamilies derived from AluY, such as AluYi6, have arisen recently, therefore elements belonging to these subfamilies have not diverged extensively from the subfamily consensus sequence. It is therefore possible to identify all members of these subfamilies through identification of AluY elements with the relevant diagnostic mutations.

Although the master gene model of proliferation appears to be true for some lineages, such as AluYe [6], it cannot be true for the whole of Alu due to the presence of many currently active source genes, each of which has given rise to a "young" Alu subfamily. The most extreme alternative to the master gene model is the transposon model, where every individual Alu element is capable of producing daughter elements. The investigation of young Alu subfamilies has led to the development of models of their expansion [7-9], which fall somewhere between these two extremes. For example, it has been reported previously, using a network phylogenetic approach, that approximately 10–20% of elements within a young Alu subfamily may operate as secondary source genes [7]. It is impossible to use traditional phylogenetic reconstruction methods to infer relationships between Alu elements, as, with their very high copy number, many elements in a young Alu subfamily share a consensus sequence, therefore the relationships between these elements could not be resolved. CpG transition mutations occur at six times the rate of other types of mutations in Alu sequences due to spontaneous deamination of 5-methylcytosine to thymine [10]. This results in homoplasy, which can lead to the inference of invalid phylogenetic relationships between sequences. Through multiple alignment of 480,000 Alu sequences, which were divided into groups based on the overrepresentation of individual mutations, it has been estimated that there may be at least 143 Alu source genes in total. This number would require many active elements to be present within each of the currently-defined subfamilies [11].

This study analyses the source gene composition of the AluYi and AluYh lineages of retrotransposons, and the influence of gene conversion on the mutational substructure of the subfamilies falling on these lineages. The activity of a source gene is suggested by the presence of groups of elements with shared combinations of mutations, particularly those groups with elements demonstrating presence/absence polymorphism. The presence of polymorphic elements sharing specific mutations is indicative of the activity of a secondary source element, as polymorphic elements have recently retrotransposed and are therefore unlikely to have accumulated such mutations in parallel. AluYi6 is described as an example of a subfamily which appears to possess numerous secondary source elements, and a novel subfamily, AluYh3a3, is presented as a subfamily which appears to have followed the master gene model of expansion.

## Results and Discussion

The number of source elements contributing to young Alu subfamilies appears to vary widely among lineages. The AluYh lineage appears to have split into two, which share three diagnostic mutations in addition to those of AluY. There is evidence for master gene expansion within the AluYh lineage, as mutations appear to have accumulated progressively in one subfamily. This provides further evidence that the master gene model remains consistent with the pattern of proliferation in some young Alu subfamilies. The AluYi lineage provides an alternative perspective, with evidence for multiple secondary source elements simultaneously contributing to the proliferation of the AluYi6 subfamily. This is suggested by the presence of multiple elements within a subfamily sharing a set of specific mutations, which suggests these mutations were present in the source element rather than occurring multiple times in parallel. The hypothesis is supported by the presence of polymorphic elements which share additional mutations from the AluYi6 consensus. This suggests that these mutations were present in the source gene which gave rise to these elements, rather than happening multiple times in parallel. However, using the genome trace archives to identify polymorphisms cannot conclusively determine whether or not an element which appears to be fixed is polymorphic, as individuals in which the element is absent may not be represented in the archives. It is therefore possible that the number of polymorphic elements, and therefore potentially the number of secondary source elements, has been underestimated. Gene conversion appears to have influenced the structure of mutations observed in both lineages, in one case, resulting in the inactivation of a putative master gene. These results are discussed in detail for the AluYh and AluYi lineages below.

### The AluYh lineage

#### AluYh7

**Figure 1:**
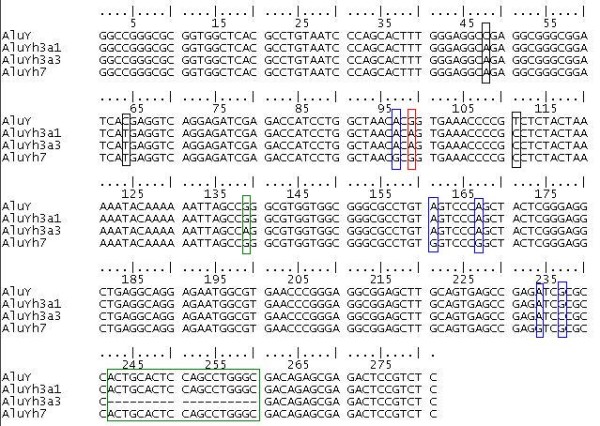
**Alignment of the AluYh7, AluYh3a1 and AluYh3a3 consensus sequences with AluY**. Diagnostic mutations from AluY can be seen for each subfamily. Mutations from AluY shared by all three subfamilies on the AluYh lineage are shown in black boxes. The further mutations accumulated by the AluYh7 subfamily are shown in blue boxes. The mutation possessed by AluYh3a1 and AluYh3a3 is shown in a red box, and the further two mutations in AluYh3a3 are shown in green boxes.

The elements of the AluYh9 subfamily [13] all share only seven diagnostic mutations, therefore this subfamily will be referred to as AluYh7 (figure 1). The subfamily is human-specific and contains 20 elements [see additional file 1], of which 16 have been previously reported as AluYh9 [13]. This subfamily appears to have arisen very recently, as at least half of the elements are polymorphic for presence or absence, and 9 of the elements are identical to the subfamily consensus. The level of divergence of the remaining 11 elements is very low, with elements possessing either 1 or 2 point mutations from the consensus. Of the two elements which possess the nine diagnostic mutations of AluYh9, one is polymorphic for presence or absence. This makes it likely that these additional two mutations are shared due to retrotransposition rather than parallel mutation or gene conversion, a non-reciprocal recombination process that affects Alu elements, so there may be two active source genes in this small subfamily.

The only evidence for proliferation on this lineage prior to the acquisition of all seven diagnostic mutations of AluYh7 is of an element with three of the seven diagnostic mutations, "AluYh3", which appears to have generated two derivative lineages, one of which is AluYh7. The second shares these three diagnostic mutations with AluYh3, along with an additional mutation (figure 1).

#### AluYh3a1

**Figure 2:**
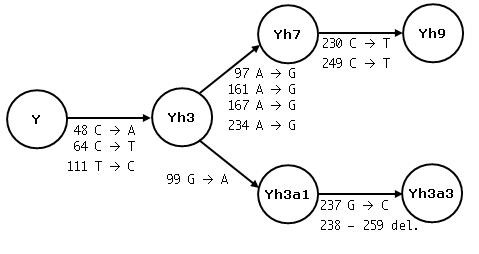
**Relationships between the subfamilies on the AluYh lineage**. Diagnostic mutations for each new subfamily are shown on the arrow leading to that subfamily. The copy numbers of each of these subfamilies are listed in table 1.

It can be assumed that this subfamily is derived from the putative "AluYh3" intermediate along this lineage (figure 2), and this subfamily will therefore be referred to as AluYh3a1, although it is possible that these three mutations from AluY occurred twice independently, this is more unlikely. All four of the diagnostic mutations for this subfamily are found in the left half of the element (figure 1). AluYh3a1 appears to have originated before the divergence of humans, chimpanzees and gorillas, as there are instances of elements of this subfamily present in the gorilla whole genome shotgun sequence. There are also instances of AluYh3a1 present in the orangutan genome, where there are at least three elements present. These elements are not found in humans. However, the subfamily appears to be absent from the available genomic data for two species of gibbon (*Hylobates concolor *and *Namascus leucogenys*). If the available gibbon sequence data are representative of the whole genome, this would suggest the AluYh3a1 subfamily originated between around 10 and 16 million years ago.

The subfamily has proliferated quite extensively in humans and chimpanzees, and many elements are shared between the two species. There are 98 elements with all four AluYh3a1 diagnostic mutations present in humans [see additional file 2], and 73 in chimpanzees [see additional file 3].

**Figure 3:**
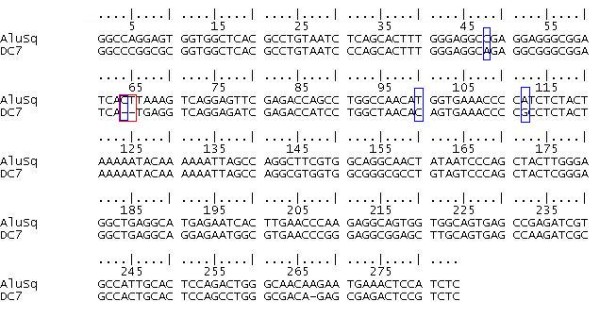
**Alignment of the chimpanzee AluYh3a1 element DC7 and the AluSq element present at the orthologous position in the human genome**. Diagnostic positions for AluYh3a1 are shown in blue boxes, the characteristic deletion of AluSq is shown in a red box. This case represents an example of complete gene conversion replacing an Alu element from an old subfamily with one from a younger subfamily.

A complete gene conversion event has occurred in chimps, where there is an AluYh3a1 present in the chimpanzee (DC7), but an older AluSq element is found at the orthologous locus in the human genome (figure 3). This is likely to be a forward gene conversion event in the chimpanzee rather than a backwards event in the human due to the high similarity between DC7 and the shared element DC8/DY83, which is likely to have provided the template. There are other examples of possible complete gene conversion events occurring between species-specific Alu elements, where pairs of elements share numerous mutations. However, as the putative gene conversion events would be occurring between two species-specific elements, it cannot be proven that the mutations are not shared due to parallel mutation.

**Figure 4:**
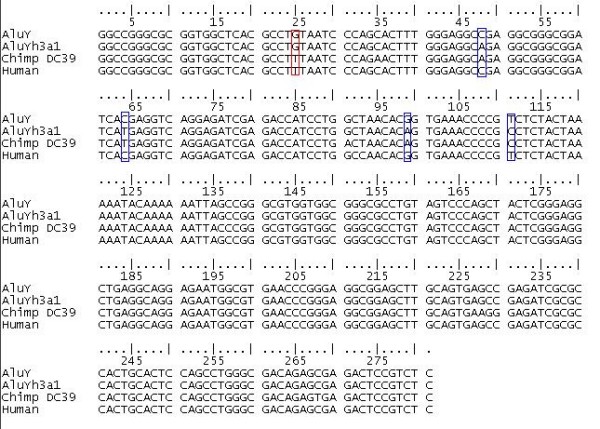
**Alignment of chimpanzee AluYh3a1 element DC39 and the human orthologue**. A partial gene conversion event has introduced diagnostic mutations into the human element, shown in blue boxes. A transversion mutation is shared between orthologues outside the putative gene conversion tract, shown in the red box. It is possible that one of these elements has been introduced by complete gene conversion, with subsequent parallel mutation at position 25.

In addition, patterns of mutations suggest multiple partial and "almost complete" gene conversion events have occurred. Comparison of elements shared by humans and chimpanzees reveals that ancestral nucleotides have been introduced at diagnostic positions in one species. This may be either due to partial gene conversion or back mutation. In two cases, all four diagnostic sites possess the ancestral nucleotide in one species, but this is likely to be due to partial gene conversion, rather than complete, as the orthologues share mutations outside the putative gene conversion tract (figure 4). In the case of AluYh3a1, the diagnostic mutations are clustered within a 64 bp region. It is therefore reasonable that a partial gene conversion tract, which on average cover around 50–100 bp [2,18], would result in ancestral nucleotides being introduced at all four sites.

There are further examples of likely partial gene conversion events resulting in between 1 and 3 diagnostic mutations. Once again, mutations are shared between orthologues on either side of the putative tract, but no mutations are shared within it.

There is no compelling evidence from the sequence data for this subfamily to suggest that secondary source elements have contributed to its proliferation. The greatest number of elements sharing a particular mutation within a species is 14 of the 98 elements. However, this mutation is a CpG transition, which is likely to have occurred many times in parallel. The 98 sequences in this subfamily differ from the consensus sequence by 2.622 mutations, on average, at non-CpG sites. This represents a difference of 0.01107 mutations per base. If we assume that CpG sites are six times as likely to change as are non-CpG bases, then, using the binomial distribution, we calculate that the probability that a given CpG base in the sequence would be mutated in 14 or more of the sequences is 0.532%. We assume that effectively all these mutations would be the C to T and the G to A changes associated with CpGs. There are 44 CpG bases in the sequence, each of which has a chance of 0.532% of being mutated in 14 or more of the sequences. Thus, we calculate that the probability that at least one of these 44 would be mutated in 14 or more of the 98 sequences is approximately 44 times this, or more than 20%. Thus this site mutated in 14 sequences is consistent with a hypothesis of 14 independent mutational events. Given the relatively small size of this subfamily, however, this may be indicative of a secondary source element. The greatest number of elements sharing a pair of mutations is 5, which is, again, possibly due to the presence of a secondary source element possessing these two mutations, but could be due to parallel mutation. For this reason, levels of polymorphism were not assessed for this subfamily.

#### AluYh3a3

There is a small subfamily which appears to have been derived from AluYh3a1 (table 1), but beyond this, there is no evidence for substructure within the AluYh3a1 subfamily to indicate the activity of further secondary source elements. Therefore, it is possible that the remaining elements in this subfamily have been produced by the activity of a single source (master) gene. Alternatively, there may be several source elements, which do not possess mutations, or perhaps only a single CpG mutation, which alone would not provide enough evidence to suggest the activity of a secondary source gene.

**Table 1:** Subfamilies on the AluYh lineage.

Putative subfamily	Species	Mutations from AluYh3 consensus	Copy number	Polymorphic?
Yh9	H	97G, 161G, 167G, 230T, 234G, 249T	2	Y

Yh7	H	97G, 161G, 167G, 234G	20	Y

Yh3a1	H, C, G, O	99A	98 (H), 73 (C)	-

Yh3a3	H, C	99A, 237C, (238–259 del.)	3 (H), 11 (C)	UN

Copy number of the AluYh7 subfamily includes elements with the diagnostic mutations for AluYh9. H = human, C = chimp, G = gorilla, O = orangutan, UN = unknown, Y = yes. Copy number in the gorilla and orangutan genomes is unknown due to the absence of complete genome sequences for these species.

The derivative subfamily, named AluYh3a3, contains a characteristic 19 bp deletion near the 3' end, between positions 242 and 260. This subfamily is very small, comprising only three elements in humans [see additional file 4], and 11 elements in chimpanzees [see additional file 5]. However, large deletions in Alu elements are rare and so the presence of the deletion in these elements, in addition to the four diagnostic mutations of AluYh3a1, is good enough evidence to consider this a unique subfamily, rather than due to parallel deletion. In addition to the 19 bp deletion, 10 of the 11 chimpanzee elements also contain a diagnostic point mutation. Two of the elements are shared between chimpanzees and humans.

**Figure 5:**
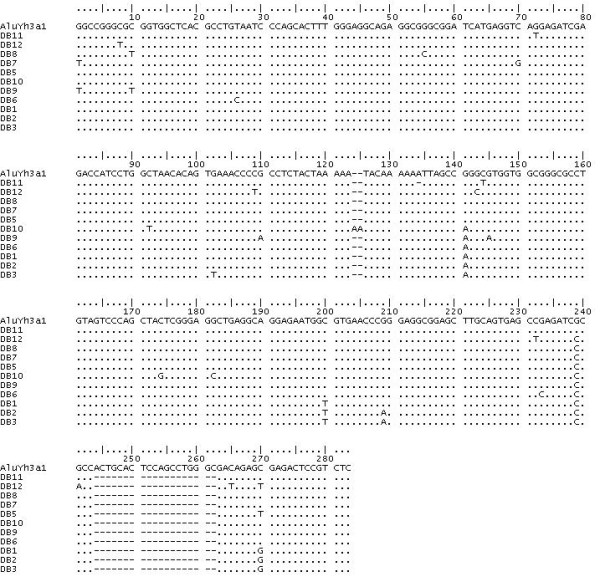
**Alignment of AluYh3a3 elements in the chimpanzee, with the AluYh3a1 consensus**. A progressive accumulation of mutations can be seen in the putative master gene, DB3, supporting the master gene model of proliferation of this subfamily.

**Figure 6:**
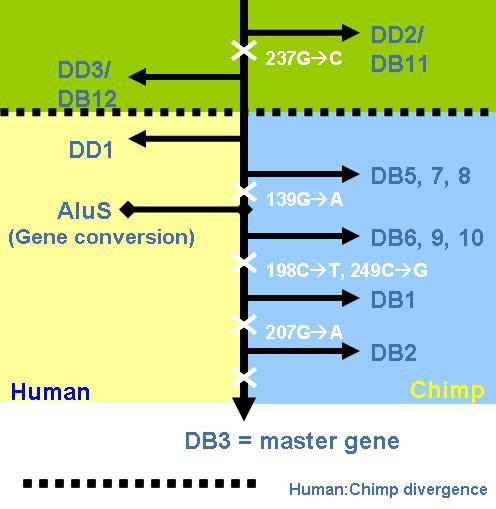
**Progressive accumulation of mutations in the AluYh3a3 master gene**. The master gene model of proliferation accounts for the sharing of mutations by several groups of elements in the AluYh3a3 subfamily in chimpanzees. A complete gene conversion event appears to have inactivated the master gene along the human lineage.

Although AluYh3a3 consists of a very small number of elements, there is a considerable amount of substructure in this subfamily. The pattern of shared mutations can be explained under the master gene model, without the inference of secondary source elements, as there appears to have been a progressive accumulation of mutations (figure 5). The putative master element would be either DB2 or DB3, as these two elements possess all of the shared mutations. However, neither of these two elements is shared between chimpanzees and humans. The orthologous locus in humans is a perfect unfilled site for DB2, showing a single copy of the TSD. The orthologous locus in humans to chimp DB3 is a filled site containing an AluSx element. It is therefore possible that DB3 is the master gene for the AluYh3a3 subfamily, but the locus has undergone backwards gene conversion in humans (figure 6). Gene conversion has been reported to be more likely between spatially close Alu elements [19], and the human DB3 orthologue is found in a region with numerous highly similar AluSx elements (possessing the characteristic 20 bp deletion) in the vicinity that could have provided the gene conversion template. Presence of this deletion might have made gene conversion more likely between this AluSx element and AluYh3a3, which contains a 19 bp deletion, as the sequences would be of similar length.

Inactivation of the master gene by gene conversion would also explain why there are fewer elements of this subfamily in humans compared with chimpanzees. This is more likely to be a backwards gene conversion on the human lineage rather than a forward gene conversion in the chimpanzee, as the site is unfilled in the orangutan genome. AluSx currently mobilizes at only very low frequency [20], and therefore is more likely to have been introduced to this locus in humans by gene conversion rather than retrotransposition.

AluYh3a3 is also present in the gorilla, however, the orthologous locus to chimp DB3 is unavailable to determine whether or not this is the original founder element. There are four copies of AluYh3a3 in the available gorilla genomic data, all of which are gorilla-specific.

No AluYh3a3 elements appear to be polymorphic in either humans or chimpanzees by examination of the trace archives. This may indicate that the subfamily is no longer proliferating, or individuals with polymorphic elements may not be represented in the archives. This is more likely for the chimpanzee sequences, as fewer individuals have been sequenced.

#### AluYi6

**Figure 7:**
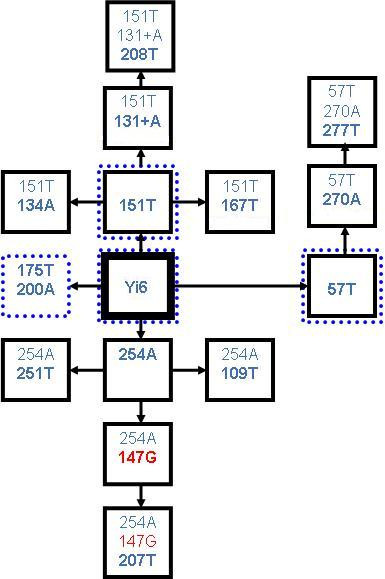
**Inferred relationships between AluYi6 derivative subfamilies**. Diagnostic mutations for each putative subfamily are shown in each box. The copy numbers of each of these putative subfamilies are listed in table 2. Blue dotted lines indicate the presence of a subfamily in chimpanzees. The mutation shown in red is a back mutation to the ancestral nucleotide at an AluYi6 diagnostic site.

The AluYi6 subfamily, which has six diagnostic mutations from the AluY consensus, has been reported to be present in humans, chimpanzees and gorillas [14]. 123 elements belonging to this subfamily have been published, 104 of which possess all six diagnostic mutations [14]. In this study, 237 Yi6 elements were identified in humans [see additional file 6]. The sequences of these elements show patterns of shared mutations consistent with the activity of potentially many secondary source elements. 3 derivative subfamilies, designated Yi6.1, Yi6.2 and Yi6.3 have already been reported, and have all been shown to be polymorphic, and therefore currently active [14]. The pattern of shared mutations in the AluYi6 subfamily indicates there may be as many as 14 source elements operating in humans (table 2). These potential 14 source genes fall on only 3 lineages, as each possesses one of three mutations: 151T, 57T or 254A (figure 7). Polymorphism data suggest that this isn't indicative of three "master genes", but does indeed represent the activity of many source elements. Some of these small Yi6 derivative subfamilies contain a considerable number of elements (for example, there are 36 elements with the 57T mutation), whereas others contain very few. The potential derivative subfamilies which only contain very few elements, such as those with 254A and 251T mutations (5 elements), may not be the product of secondary source genes, as the mutations may simply be shared due to parallel mutation. Polymorphism data, however, suggest the former is more likely.

**Table 2:** Putative AluYi6 derivative subfamilies.

Putative subfamily	Species	Mutations from Yi6 consensus	Copy number	Polymorphic?
Yi6	H, C, G	-	237 (H), 91 (C)	Y

Yi6.1	H, C	57T	36 (H), 7 (C)	Y

Yi6.1a	H	57T, 270A	10	Y

Yi6.1b	H	57T, 270A, 277T	4	Y

Yi6.2	H, C	151T	77 (H), 17 (C)	UN

Yi6.2a	H	151T, 134A	8	Y

Yi6.2b	H	151T, 167T	5	UN

Yi6.2c	H	151T, 131+A	53	Y

Yi6.3	H	151T, 131+A, 208T	22	Y

Yi6.4	H	254A	35	UN

Yi6.4a	H	254A, 251T	5	UN

Yi6.4b	H	254A, 109T	3	UN

Yi6.4c	H	254A, 147G	20	UN

Yi6.4d	H	254A, 147G, 207T	18	Y

Yi6.5	C	175T, 200A	31	Y

Yi6.1, Yi6.2 and Yi6.3 reported in [14]. Where a shared mutation is found with additional shared mutations, the copy number of elements with the single mutation includes copies with further shared mutations. H = human, C = chimp, G = gorilla, UN = unknown, Y = yes.

It was shown previously that three of the elements found in humans were shared with the chimpanzee [14]. Analysis of the chimpanzee genome reveals that Yi6 has proliferated quite extensively in the chimpanzee following its divergence from humans. 91 Yi6 elements were found to be present in the chimpanzee genome [see additional file 7], of which 13 are shared with humans. Only three of the chimpanzee elements are identical to the AluYi6 subfamily consensus. Two of the previously identified derivative subfamilies (Yi6.1 and Yi6.2) were found in both humans and chimps, suggesting a time of origin prior to the human-chimp divergence. A novel chimp-specific subfamily was also identified, the consensus for which has two additional diagnostic mutations (175A, 200A) relative to the AluYi6 consensus. 31 of the 91 chimpanzee Yi6 elements belong to this novel subfamily. In chimpanzees, at least three AluYi6 source genes appear to be currently active from looking at polymorphism data, containing the 151T mutation, the 175A and 200A mutations, and the 57T mutation.

Two of the elements that were found to be shared between humans and chimps in the original study were also found in the gorilla [14], suggesting the subfamily arose before the divergence of gorillas from chimpanzees and humans, approximately seven million years ago. Yi6 does appear to have undergone some proliferation along the gorilla lineage, with at least one gorilla-specific element present in this species. AluYi6 appears to be absent from the orangutan, with no evidence of the subfamily in the orangutan pre-ensembl shotgun assembly. This suggests the subfamily is less than 10 – 13 million years old [21,22].

Gene conversion, a non-reciprocal recombination process commonly affecting Alus, has also operated in the AluYi6 subfamily. There is evidence for a complete gene conversion event, as there is an AluYi6 element present in the chimp (DQ59), and an older Alu belonging to the AluY subfamily present at the orthologous locus in humans.

### Difference in retrotransposition rate between humans and chimpanzees

The rate of retrotransposition has been shown to have increased along the human lineage since the divergence between humans and chimpanzees [23], with most young Alu subfamilies that are present in both species reaching larger copy numbers in humans than chimpanzees. The observations of copy number of subfamilies in the AluYh and AluYi lineages generally support this, with subfamilies reaching a higher copy number in humans than chimpanzees where they are present in both species. For example, out of the 73 AluYh3a1 elements found in chimpanzees with all four diagnostic mutations, only 16 are unique to chimpanzees, with the remaining 57 found in both chimpanzees and humans. Out of the 98 human elements, 41 are human-specific. In addition, fewer copies of AluYi6 are found in chimpanzees relative to humans. AluYh3a3 is unusual in that it has proliferated to a greater extent in chimpanzees than humans. However, this has been shown to be due to inactivation of the master gene along the human lineage some time into the past, thereby preventing further proliferation of the subfamily.

#### AluYg6

AluYg6, a subfamily previously investigated in detail (Styles and Brookfield, 2007), was also assessed for the presence of polymorphic elements which might indicate the activity of secondary source genes (table 3). Polymorphic elements belonging to the derivative subfamilies Yg6a2 and Yg5b3 were identified, revealing that these subfamilies are at least 30% and 26% polymorphic, respectively. Polymorphic elements matching the AluYg6 consensus sequence was also identified. In addition to polymorphic elements with the Yg6a2 and Yg5b3 consensus sequences, polymorphic elements were identified with the diagnostic mutations for each of these subfamilies along with an additional shared mutation in each case. Three of the five AluYg6a2 elements, and two out of seven of the AluYg5b3 elements, with the additional mutation, were found to be polymorphic. The presence of polymorphic elements with additional shared mutations suggests that the number of source elements in the AluYg6 subfamily is higher than previously thought, and may be at least 5.

**Table 3:** AluYg6 derivative subfamilies.

Subfamily	Species	Mutations from Yg6 consensus	Copy number	Polymorphic?
Yg6	H, C	-	380 (H) 1 (C)	Y

Yg6a2	H	153T, 174A	40	Y

Yg5b3	H	94G, 172T, 246G	27	Y

Copy number of the AluYg6 subfamily includes elements belonging to the two derivative subfamilies. H = human, C = chimp, Y = yes.

## Conclusion

It is clear that there is considerable variation in the number of source genes present in each of the young Alu subfamilies. Evidence from patterns of shared mutations and polymorphism data suggest that multiple source genes are actively retrotransposing in the AluYh7 and AluYi6 subfamilies, the latter of which may contain up to 14 source elements. The data presented here support the hypothesis that neither the master gene model nor the transposon model is valid for all young Alu subfamilies. There is not sufficient evidence to suggest the presence of secondary source genes contributing to the proliferation of AluYh3a1. The small AluYh3a3 subfamily appears to have followed the master gene model of proliferation in both humans and chimpanzees, with its substructure easily explained without the need to infer the activity of secondary source elements. Polymorphism data suggest the presence of several secondary source genes contributing to the proliferation of the AluYg6 subfamily. Gene conversion appears to have operated in the AluYh3a1, AluYh3a3 and AluYi6 subfamilies, with partial gene conversion introducing ancestral mutations at diagnostic sites, and both forward and backward complete gene conversion replacing Alu elements with those belonging to other subfamilies. In the case of AluYh3a3, such an event has resulted in inactivation of the putative master gene in humans.

## Methods

### Sequence data

A BLASTN search [12] of the human genome sequence, using default parameters, was performed using the consensus sequences of the AluYh9 [13] and AluYi6 [13,14] subfamilies, obtained using RepBase Update [15]. The searches yielded 1426 and 1277 Alu elements, respectively, which were manually examined for the presence of diagnostic mutations specific to AluYh9 and AluYi6. Those which possessed the diagnostic mutations of AluY, and at least seven of the additional diagnostic mutations of the AluYh9 subfamily, and at least four of those for AluYi6, were extracted. It was not required that elements possessed all diagnostic mutations for each subfamily, as back mutation can introduce ancestral nucleotides at diagnostic sites. In each case, following the extraction of elements with the necessary diagnostic mutations, the remaining hits in the BLAST search corresponded to AluY elements. The search using the AluYh9 consensus revealed only two elements with all nine diagnostic mutations, with the majority of elements sharing only seven. This subfamily will be referred to as AluYh7. Many Alu elements were identified using the AluYh7 consensus sequence, which shared three of the diagnostic mutations of the subfamily, and an additional point mutation. These elements will be referred to as AluYh3a1 (figure 1). This sequence was then used as a query for a BLAST search. Elements from the AluYh7, AluYh3a1, and AluYi6 subfamilies were extracted. A custom Perl program was used to identify any mutations that had occurred in each element relative to the corresponding subfamily consensus.

1000 bp of flanking DNA was extracted 5' and 3' of each element, to enable the identification of orthologous regions in closely-related species. Each of the extracted elements and its flanking sequence were submitted as a query for a BLAT [16] analysis of a related genome (chimpanzee or human) to elucidate complete gene conversion events. Orthologous regions were aligned using ClustalW [17]. In most cases, a gap was present in one species corresponding to the region in which the Alu, and one copy of the target site duplication (TSD), were found in the other species. In some cases, an Alu element of an older subfamily was present at the orthologous position, indicative of a gene conversion event.

### Polymorphism

The NCBI human and chimpanzee genome trace archives were used to assess whether or not each element is polymorphic for presence or absence. This can be seen where the flanking DNA is present in the trace archive, with only one copy of the TSD, and the corresponding Alu is absent.

## Abbreviations

bp: base pairs; mya: million years ago; TSD: target site duplication.

## Authors' contributions

PS identified the sequences and carried out the alignments and evolutionary interpretations and wrote the majority of the manuscript. JFYB suggested the underlying approach, carried out statistical calculations, and helped to draft the manuscript. Both authors read and approved the final manuscript.

## Supplementary Material

Additional file 1 **Alignment of AluYh7 elements in humans**. An alignment of all AluYh7 elements present in the complete human genome sequence. Click here for file

Additional file 2 **Alignment of AluYh3a1 elements in humans**. An alignment of all AluYh3a1 elements present in the complete human genome sequence. Click here for file

Additional file 3 **Alignment of AluYh3a1 elements in chimpanzees**. An alignment of all AluYh3a1 elements present in the complete chimpanzee genome sequence. Click here for file

Additional file 4 **Alignment of AluYh3a3 elements in humans**. An alignment of all AluYh3a3 elements present in the complete human genome sequence. Click here for file

Additional file 5 **Alignment of AluYh3a3 elements in chimpanzees**. An alignment of all AluYh3a3 elements present in the complete chimpanzee genome sequence. Click here for file

Additional file 6 **Alignment of AluYi6 elements in humans**. An alignment of all AluYi6 elements present in the complete human genome sequence. Click here for file

Additional file 7 **Alignment of AluYi6 elements in chimpanzees**. An alignment of all AluYi6 elements present in the complete chimpanzee genome sequence. Click here for file
